# The effect of inorganic salt on the morphology and nucleation of polyaniline nanofibers synthesized via self-assembly

**DOI:** 10.1080/15685551.2023.2166727

**Published:** 2023-01-18

**Authors:** Ruijuan Wang, Yiqi Jing

**Affiliations:** Guangxi Key Lab of Agricultural Resources Chemistry and Biotechnology, Schoolof Chemistry and Food Science, Yulin Normal University, Yulin, P. R. China

**Keywords:** Inorganic salt, PANI nanofibers, the nucleation, in situ UV-vis spectroscopy, in situ ^1^H NMR

## Abstract

Polyaniline (PANI), due to the various and controllable shapes, the environmental stability, the excellent physical and chemical property, has gained significant attention. PANI with abundant morphologies were successfully prepared through adjusting and controlling the state of the initial micelle-like in the micelle-like system composed by aniline and organic acids with relatively weak intermolecular interaction. Although the influence of the inorganic salts on their morphology, including the surface and the diameter, was investigated, the influence of salt on the nucleation of PANI was still unclear. Therefore, PANI nanofibers were fabricated through the addition of inorganic salt such as NaCl, MgSO_4_ and AlCl_3_ into the micelle-like composed of aniline and D-camphor-10-sulfonic acid. The influence of types and concentration of inorganic salts, doped acids and temperature on PANI was studied by Transmission Electron Microscope (TEM), UV-vis and Fourier Transform Infrared Spectroscopy (FTIR) spectroscopy. In addition, in situ UV-vis and ^1^H Nuclear Magnetic Resonance technology (NMR) were applied to observe the process of aniline polymerization, and it was indicated the polymerization rate of aniline changed after the addition of inorganic salt NaCl into the initial solution.

## 1 Introduction

Polyaniline (PANI) and its composites have attracted lots of attention in various fields such as adsorption [1–3], anticorrosion [4–6], cathode materials [7–9] and sensors [10–12], due to abundant morphologies, simple preparation, excellent environmental stability, unique physical and chemical property, and high electrochemical property. Nanostructured PANI with abundant shapes have been synthesized in micelle-like system [13–17], but many factors, such as ionic strength [18–20], the types and the concentration of doped acid [21], temperature [22], and the external field [23–25], affect the morphology and the property of the product in micelle-like system. At present, inorganic salts as the additives have been applied to prepare nanostructured PANI. Zhang and his team [18] successfully prepared chrysanthemum flower-like PANI composed of nanofibers with high specific capacitance and high crystallinity through the addition inorganic salts via a self-assembly process; Pahovnik and his college [19] synthesized PANI with different nanostructures in the presence of inorganic electrolyte and organic electrolyte; Liu and his coworker [20] synthesized PANI nanosheets with good cycling stability and high specific capacitance in saturated NaCl solution with HCl as doped acid. The effect of inorganic salt on the morphology and electrochemical property has been investigated, but the effect of inorganic salt on the nucleation of PANI was not clear. Besides, in situ UV-vis [26] and ^1^H NMR [27] observation have been applied into the nucleation mechanism of nanostructured PANI, the evolution information of oligomers can be obtained from in situ observation during nucleation stage.

Hence, PANI nanofibers were synthesized in the micelle-like system composed by aniline and camphor sulfonic acid in this study, and in situ UV-vis spectroscopy and in situ ^1^H NMR were executed to investigate the effect of inorganic salt on the polymerization process of aniline.

## 2 Experimental Section

### 2.1 Chemical Reagents

Aniline and acetone were bought from Shanghai Lingfeng Chemical Reagent Co. Ltd; Ammonium persulfate (APS), N-Methyl-2-pyrrolidone (NMP) and D-camphor-10-sulfonic acid (D-CSA) were obtained from Sinopharm Chemical Reagent Co. Ltd; NaCl and ethanol were purchased from Nanjing Chemical reagent Co. Ltd; AlCl_3_⋅6H_2_O was obtained from Xilong Chemical Co. Ltd; MgSO_4_ was bought from Shanghai shisihewei Chemical Co. Ltd and Shanghai Qiangshun chemical Co. Ltd, respectively; the distilled water was from the lab. Aniline was distilled with reduced pressure before usage.

### 2.2 Preparation of PANI

#### 2.2.1 Synthesis of PANI in the absence of D-CSA

A series of aqueous solution with 0.086 mol/L aniline and 0.2 mol/L inorganic salts (NaCl, MgSO_4_ and AlCl_3_) were prepared, and kept standing for 24 h at 5°C; and then, the equivalent APS solution as the oxidant was quickly added into the above solution to initiate polymerization; after 24 h, the samples were washed with different solvent such as water, ethanol and acetone, dried for 12 h at 60°C, and finally collected.

#### 2.2.2 Synthesis of PANI in the presence of D-CSA

A series of aqueous solution with 0.086 mol/L aniline, 0.086 mol/L D-CSA and 0.2 mol/L inorganic salts, and the subsequent steps were the same as above-mentioned; and the samples were collected in final.

### 2.3 Characterizations

The morphology of the samples was characterized through transmission electron microscopy (JEM-2100); A small amount of PANI was ultrasonically dissolved in NMP for 5 min, and the UV-vis spectra of the solution was recorded by UV-vis spectrometer (Cary 5000); the structures of PANI were measured through FTIR (Bruker Vector 22); a series of in situ UV-vis spectra and ^1^H NMR spectra were used to monitor the evolution information of the oligomers in the polymerization process of aniline, and the CHI660B electrochemical workstation was applied to measure the electrochemical properties of PANI, cyclic voltammetry and galvanostatic charge-discharge were performed through three electrodes system in 1.0 M H_2_SO_4_ electrolyte.

## 3 Results and Discussions

### 3.1 The influence of D-CSA and inorganic salts on PANI

**Figure 1.**
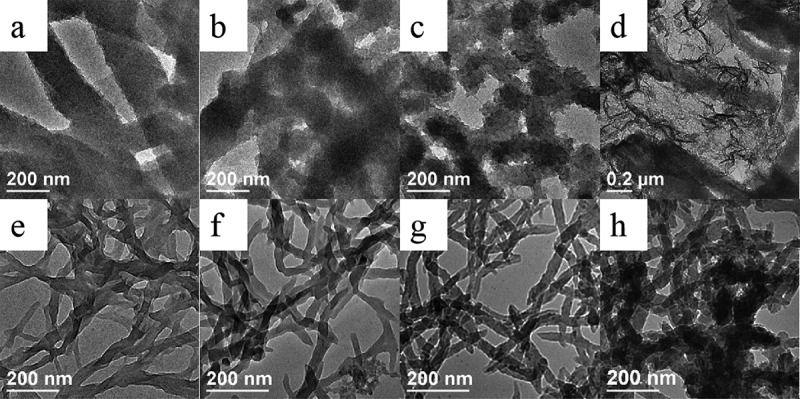
TEM images of PANI synthesized in the absence (a–d) and presence (e–h) of D-CSA under the different inorganic salts: (a, e) no salts; (b, f) NaCl; (c, g) MgSO_4_ and (d, h) AlCl_3._

Figure 1 displays the morphology of PANI synthesized at the different conditions. For the initial systems in the absence of D-CSA, the shape of PANI prepared without inorganic salts was nanofiobers with rough surface and the diameter of 169 nm in Figure 1a; when inorganic salts (NaCl, MgSO_4_ and AlCl_3_) was introduced into the initial solution, the morphology of as-synthesized PANI was still nanofiber with rough surface, and the average diameter of the as-synthesized PANI nanofibers in Figure 1b–1d was 158, 109 and 172 nm, respectively. For the initial system containing D-CSA, the morphology of PANI prepared without salts was nanofibers with smooth surface and the average diameter of 30 nm in Figure 1e; when NaCl, MgSO_4_ and AlCl_3_ was added into polymerization, the morphology of PANI was still nanofibers in Figure 1f–1h, the diameter of PANI nanofibers with the smooth surface was 25, 32 and 42 nm, respectively. Compared with the system without D-CSA, the diameter of PANI nanofibers prepared in the presence of D-CSA remarkedly reduced, suggesting that the addition of D-CSA was helpful to obtain the fibers with small diameter and smooth surface. Hence, the addition of D-CSA into the initial solution was used to prepare PANI nanofibers. Besides, the introduction of NaCl contributed to the decrease of the diameter of PANI nanofibers to a certain extent.

**Figure 2.**
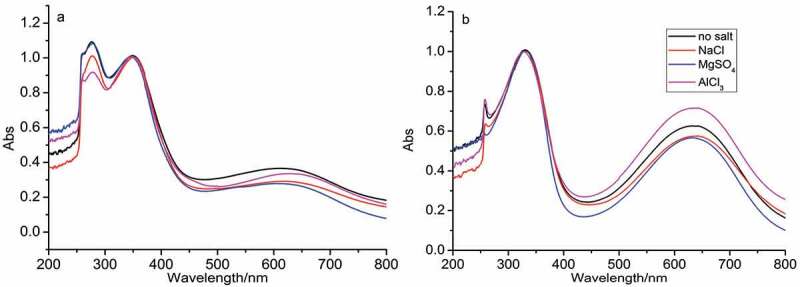
UV-vis spectra of PANI synthesized in the absence (a) and presence (b) of D-CSA.

**Figure 3.**
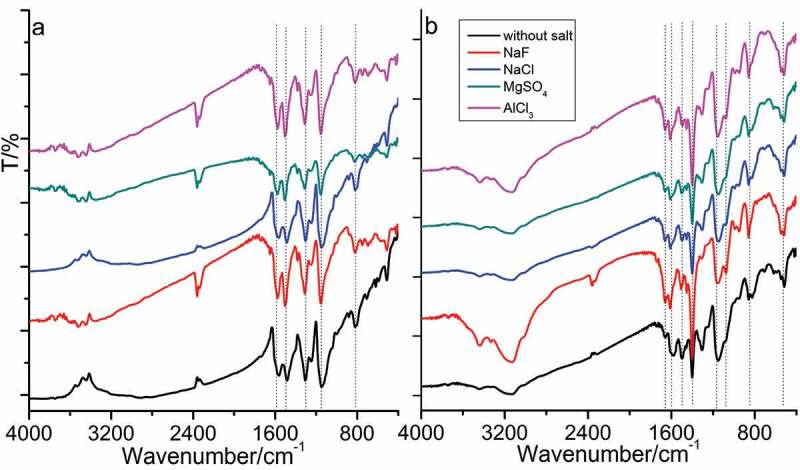
FTIR spectra of PANI synthesized in the absence (a) and presence (b) of D-CSA.

Figures 2 and 3 show the UV-vis spectra and FTIR spectra of as-synthesized PANI in the absence and presence of D-CSA. As Figure 2 shows, the similar peaks of PANI synthesized the initial system without D-CSA in the absence of salts and in the presence of NaCl, MgSO_4_ and AlCl_3_ were observed at 270 and 370 nm, resulting from π-π* tranisition in pH-neutral PANI molecule containing both quinoid and benzenoid units [28], and the broad peak at 650 nm was attributed to excitonic-type transition between HOMO orbital of the benzenoid ring and LUMO orbital of the quinoid ring, resulting from the participation of sulfuric acid produced by hydrolysis of APS oxidant into the polymerization. For the initial system including D-CSA, the similar peaks of PANI produced with inorganic salts occurred at 330 and 650 nm in Figure 2b.

The characteristic peaks of PANI without D-CSA observed were similar in FTIR spectra, as Figure 3a shows. The peaks located at 1598 and 1486 cm^−1^ were attributed to the stretching vibration of C=C on quinone ring and benzene ring, respectively; the peak at 1313 cm^–1^ corresponded to the stretching vibration of C–N on aromatic amines from secondary structure; the peak at 1161 cm^−1^ was attributed to the bending vibration of aromatic C–H; the peak at 833 cm^−1^ was caused by the out of plane deformation of C–H on 1,4-disubstituted benzene ring [24]. When the organic acid D-CSA was added into the polymerization, the characteristic peaks of PANI salt shifted to 1618, 1598, 1498, 1397, 1168 and 858 cm^−1^ in Figure 3b, in agreement with the reported literature [23]. In addition, the new peaks were observed, such as the peaks located at 1068 and 697 cm^−1^ were the absorption characteristic peak of –SO_3_ group; the peaks at 528 cm^−1^ belonged to the stretching vibration of S–O group [23].

### 3.2 The influence of the concentration of inorganic salt and the temperature on PANI

**Figure 4.**
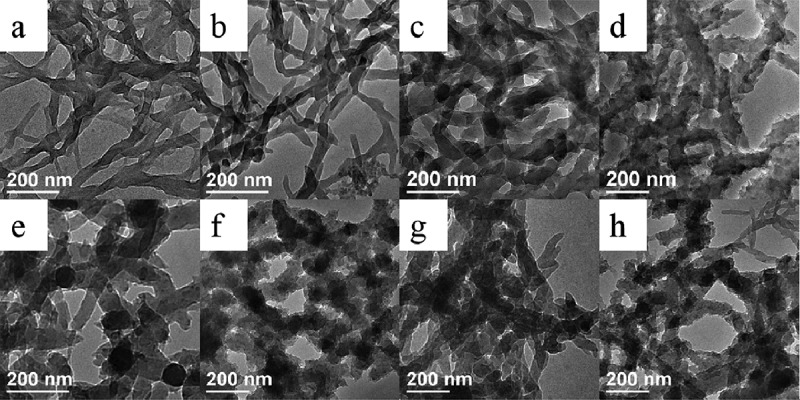
TEM images of PANI prepared at 5°C (a–d) and 25°C (e–h) at different concentration of NaCl: (a, e) 0 M; (b, f) 0.2 M; (c, g) 0.4 M and (d, h) 0.6 M NaCl.

**Figure 5.**
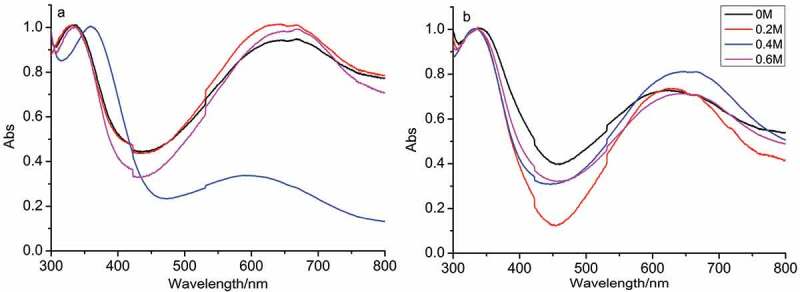
UV-vis spectra of PANI prepared at different concentration of NaCl at 5°C (a) and 25°C (b), and [NaCl] = 0; 0.2; 0.4 and 0.6 M.

The introduction of NaCl has similar effect on the diameter of PANI nanofibers synthesized with or without D-CSA on the basis of the abovementioned analysis. Therefore, NaCl was selected to investigate the effect on the shape and structure of PANI by changing the concentration of NaCl. Meanwhile, to investigate the influence of the temperature on PANI, the temperature (5°C and 25°C) was applied to prepare PANI. A series of solution including 0.086 mol/L aniline and 0.086 mol/L D-CSA were prepared, and the amount of NaCl was added into the solution; the mixture solution was kept standing for 24 h, and then the polymerization was triggered by APS solution and reacted for 24 h at the different temperature. Figures 4 and 5 display TEM images and UV-vis spectra of as-synthesized PANI. As Figure 4 shows, the morphology of PANI fabricated at 5°C and 25°C was still nanofiber, but the diameter of PANI nanofibers synthesized at the different temperature was different. When the concentration of NaCl was less to 0.2 M, the diameter of PANI nanofibers synthesized at 5°C reduced and their surface became smooth, while the diameter of PANI nanofibers fabricated at 25°C increased from 56 to 62 nm and the surface became rough; as the concentration of NaCl continued to increase, PANI nanofibers synthesized at 5°C and at 25°C became large and rough. The probable reason was: the low nucleation and growth ratio of PANI at the low temperature resulted in a decrease in the diameter of PANI nanofibers, compared with the room temperature. It was in agreement with the information obtained according to in-situ monitoring in the polymerization. Furthermore, the appropriate amount of NaCl conduced to the decrease of the diameter.

According to UV-vis spectra in Figure 5, the peaks of PANI synthesized at the different temperature occurred at 340 and 640 nm, and the ratio of the intensity of the two peaks increased as the temperature increased, which indicated the doping degree of PANI increased, when the concentration of NaCl was 0.4 M, the doping degree of PANI markedly increased, compared with that synthesized at 5°C.

### 3.3 In situ UV-vis and ^1^H NMR of polymerization process

To investigate the influence of NaCl on the nucleation of PANI, in situ UV-vis and ^1^H NMR were applied to observe the evolution of aniline polymerization. Figures S1 (in Supporting Information), 6 and S2 (in Supporting Information) displayed a series of UV-vis spectra recorded the evolution information of aniline polymerization with the different concentration of NaCl at 5°C and 25°C. The polymerization process was divided into three stages: a) the initiation stage, the peaks in the UV-vis spectra was no obvious in the first stage; b) the peak at 410 nm was observed and the intensity increased at the second stage before the broad peak occurred; c) the third stage was the growth stage, the broad peak at 600–800 nm was observed and increased in this stage. Generally speaking, the new peak at 410 nm occurred in the initial stage of polymerization, which was attributed to o-aminodiphenylamine structure produced in the stage; the broad peak was observed at 600–800 nm, which was related to the doping state and the polaron of PANI [26]; and the intensity of the two peaks increased with the polymerization time increasing, which indicated the amount of PANI increased; when the intensity increased to a certain degree and began to decrease, resulting from the precipitation of PANI in the solution. Besides, at the low temperature, the ladder phenomenon was observed at 600–850 nm, owing to the mist on the surface of the cuvette caused by the low temperature, but the spectra, especially the peak at 410 nm, can still provide us with the information of the polymerization process in Figures S1 (in Supporting Information) and 6. The detailed analysis was as follows:

**Figure 6.**
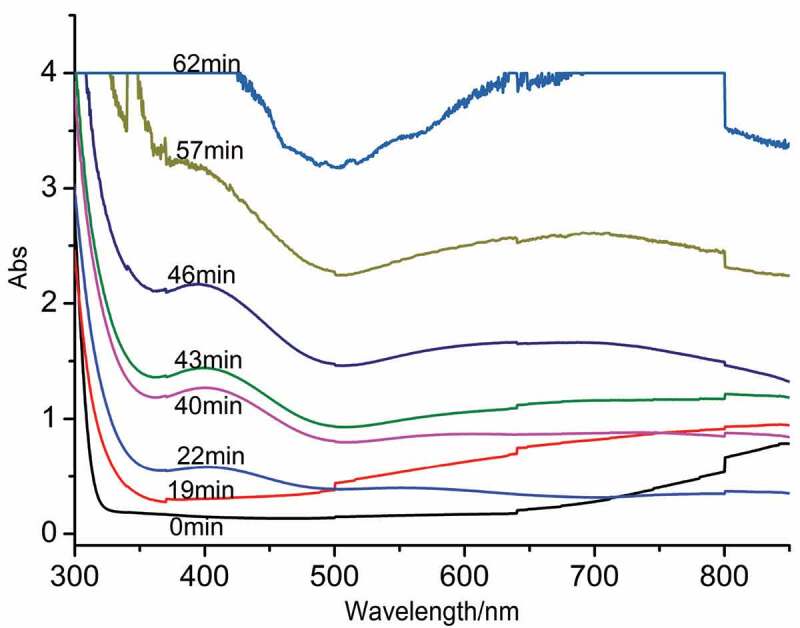
In situ UV-vis spectra of PANI prepared at 0.2 M NaCl at 5°C.

For the initial solution without NaCl, the polymerization of aniline at the different temperature was shown in Figures S1a and S2a. When the temperature was low in Figure S1a, the broad peak was newly observed at 7 min, and the intensity of the peak increased while the width of the peak reduced with the reaction time prolonging, in comparison with the peak at the different time of polymerization (10 and 22 min); when the polymerization time was 31 min, the intensity of the peak at 410 nm firstly decreased and then increased. When the temperature was room temperature in Figure S2a, the intensity of the peak at 410 nm gradually increased in the first 13 min; with the increase of the reaction time up to 19 min, the intensity of peak located at 410 nm decreased, and the broad peak at 600–800 nm occurred and increased; the peaks at 410 and 600–800 nm increased gradually. These changes suggested that the type and quantity of produced oligomers were constantly changing in the polymerization process. Therefore, the polymerization of aniline without NaCl was slow at 5°C, the reason was that the movement rate of aniline molecules and oligomers was reduced, the effective movement and collision between molecules, and the reaction speed was also reduced due to the low temperature.

When the concentration of NaCl in the solution was equal to 0.2 M at 5°C, in situ UV-vis spectra was almost no significant change in the first 19 min in Figure 6, the intensity of the peak at 410 nm was observed and gradually increased from 22 to 40 min, but the shape of the peak kept unchanged; and the intensity of the peak at 410 nm markedly increased during the next 3 min, in the meantime, the broad peak at 600–800 nm occurred, and the intensity of the broad peak increased with the increase of the polymerization time. Compared with the polymerization without NaCl at low temperature in Figure S1a, the initiation stage of polymerization process became long, which indicated the introduction of NaCl markedly reduced the reaction rate in the initiation stage.

However, according to in situ UV-vis spectra of the reaction in the presence of 0.2 M NaCl proceeded at the room temperature in Figure S2b, the peak at 410 nm gradually increased within the first 13 min, and then the broad peak at 600–800 nm occurred and increased step by step. Compared with that without NaCl at room temperature in Figure S2a, the reaction rate of polymerization was relatively slow at the initiation stage and then increased at the growth stage, different from the effect of NaCl on the polymerization at low temperature.

The polymerization of aniline in the presence of 0.4 M NaCl was displayed in Figures S1b and S2c. UV-vis spectra recorded at the low temperature in Figure S1b was similar during the first 22 min, the peak at 410 nm increased in the following 9 min and decreased from 31 to 49 min, the broad peak at 600–800 nm occurred and sharply increased at 52 min, seen in Figure S1b. Compared with those change of polymerization with the different concentration of NaCl at 5°C, the initiation stage and the growth stage of polymerization process was extended with the increase of NaCl, indicating that the introduction of NaCl was helpful to reduce reaction rate.

On the other hand, when the concentration of NaCl was 0.4 M at the room temperature, the peak at 410 nm occurred at 7 min and gradually increased, the broad peak at 600–800 nm was observed and increased at 16 min, according to the change observed in Figure S2c. In comparison with those observed in Figures S2a and S2b, the initiation stage increased, but the growth stage shortened, which suggested that the addition of NaCl was profit for the decrease of reaction rate in the initiation and the increase of the reaction rate at the growth stage.

According to the above analysis, the introduction of NaCl was in favor of the decrease of the reaction rate at the initiation stage, regardless of the low or high temperature; however, the effect of the NaCl on the reaction rate at the growth stage was different when the temperature was different, the reaction rate at the growth stage reduced when the temperature was low, while the rate increased at the room temperature.

**Figure 7.**
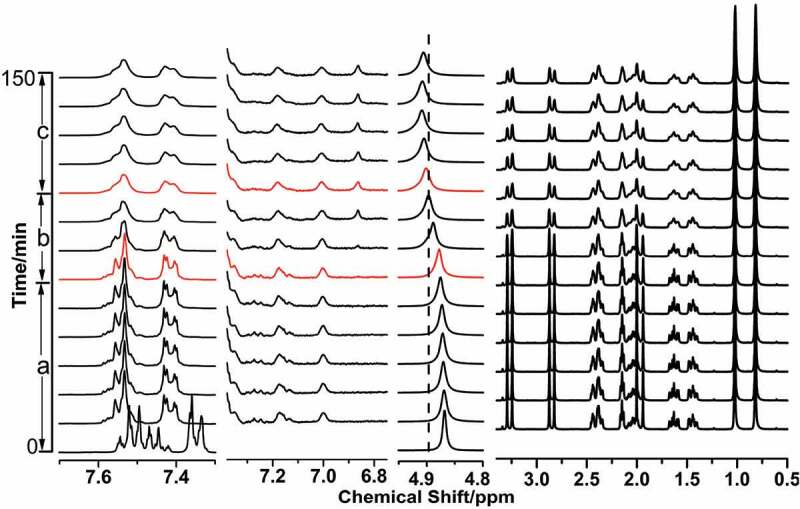
In situ ^1^H NMR spectra of PANI prepared in the micelle-like system composed of aniline and D-CSA at 5°C and the signal of D_2_O located at 4.87 ppm, a) the initiation stage after the addition of APS; b) the formative stage of phenazine structure; c) the separation of D-CSA from the micelle-like structure.

**Figure 8.**
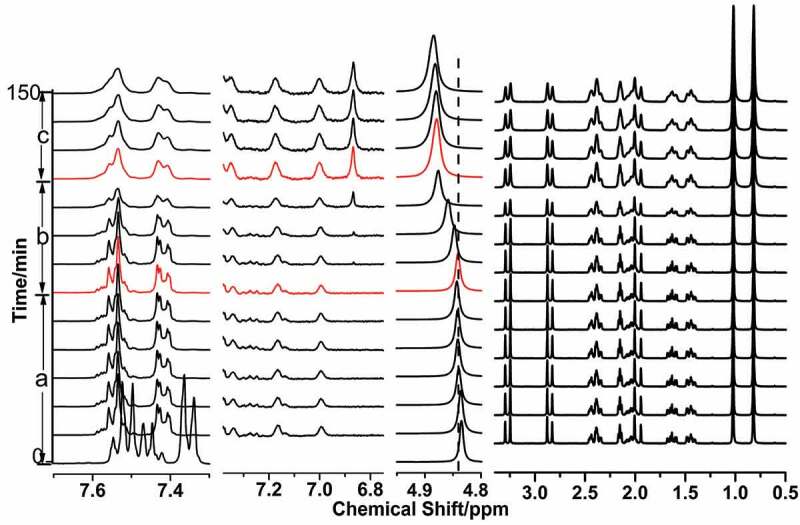
In situ ^1^H NMR spectra of PANI prepared in the micelle-like system containing 0.2 M NaCl at 5°C and the signal of D_2_O located at 4.84 ppm, a) the initiation stage after the addition of APS; b) the formative stage of phenazine structure; c) the reaction of aniline in micelle-like through outward diffusion.

In situ ^1^H NMR spectra of the evolution in the process of aniline polymerization with the different concentration of NaCl at the low temperature is shown in Figures 7 and 8, the spectra was divided into four sections, according to the signals of the species such as the anilinium, produced oligomers, water and D-CSA. When the polymerization of aniline proceeded in the absence of NaCl in Figure 7, the signals at 7.35, 7.53 and 7.45 ppm was attributed to ortho-H, meta-H and para-H of aniline in the initial solution, and the signals of D-CSA occurred during 4.0–0.6 ppm. The introduce of D-CSA had the significant influence on para-H of aniline, which was in agreement with the micelle-like system composed of aniline and salicylic acid [13,14,27], the reason was that the positive charge of anilinium cation was averaged by the aniline molecules around it, and these averaged aniline and D-CSA formed the micelle-like. According to the evolution information of the signals of aniline, oligomers and water, the polymerization of aniline was divided into three stage in Figures 7 and 8 after the addition of APS oxidant, a) the signals of anilinium occurred at 7.54 and 7.42 ppm, and the signals of the dimers produced occurred at 7.18 and 7.00 ppm in the first stage in Figure 7a, and meanwhile, the signal of water shifted downfield, and the signals of D-CSA had no obvious change; b) the signal of water moved toward downshift, and the new signal was observed at 6.87 ppm, which belonged to the para-H in phenazine structure, but the signals of anilinium cation and D-CSA kept unchanged in Figure 7b; c) the signal of anilinium cation became wide in Figure 7c, and the signal of water shift downfield, the signals of phenazine structure oligomers did not change significantly and the signals of D-CSA became weak, due to the separation of D-CSA from the micelle-like structure.

Compared with the initial solution without NaCl, the shape of the signals of aniline and D-CSA in the initial solution containing 0.2 M NaCl were no evident changes in Figure 8; the change of the peaks in the polymerization process of aniline after the addition of APS was similar in the first stage, indicating that the formative process of PANI nanofibers was similar in the absence and presence of NaCl; but the duration in the second stage was extended, consistent with the information obtained by in situ UV-vis spectra, and the signal intensity of water and phenazine oligomers gradually increased in the third stage, which suggested that the introduction of NaCl made aniline monomers in the micelle-like diffuse outward through adjusting intermolecular forces in micelle-mike system and continue to react with the aniline dimer on the micelle surface. Therefore, the relatively weak intermolecular force promoted the formation of PANI nanofiber.

### 3.4 The electrochemical properties of PANI

**Figure 9.**
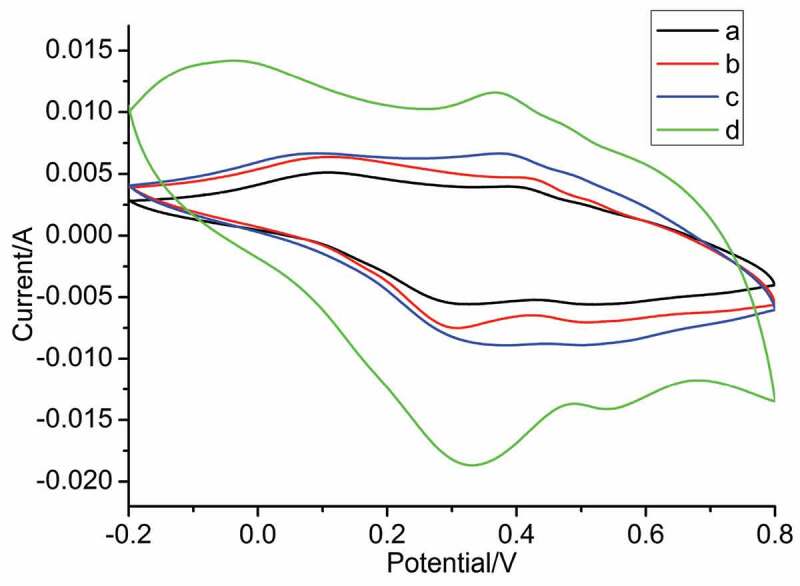
Cyclic Voltammograms of PANI prepared at the different conditions in 1.0 M H_2_SO_4_ at a scan rate of 5 mV^−1^ (vs. Saturated calomel electrode (SCE)): (a) no salt; (b) NaCl; (c) MgSO_4_ and (d) AlCl_3._

In order to investigate the effect of the introduction of inorganic salts on the electrochemical properties of nanostructured PANI, cyclic voltammetry experiments were carried out on the obtained samples, and the results are shown in Figure 9. There were two groups of redox peaks of as-synthesized PANI, due to the doping and dedoping reactions in PANI. The peaks of 0.24/0.02 V belonged to the intermediate state of PANI from the reduced state of PANI losing two electrons, and the peaks at 0.50/0.41 V corresponded to the oxidized state of PANI from the intermediate state of PANI losing two electrons. The area of cyclic voltammetry curve represents the capacitance, so it can be seen that the capacitance of as-synthesized PANI increases with the increase of ionic strength in solution.

**Figure 10.**
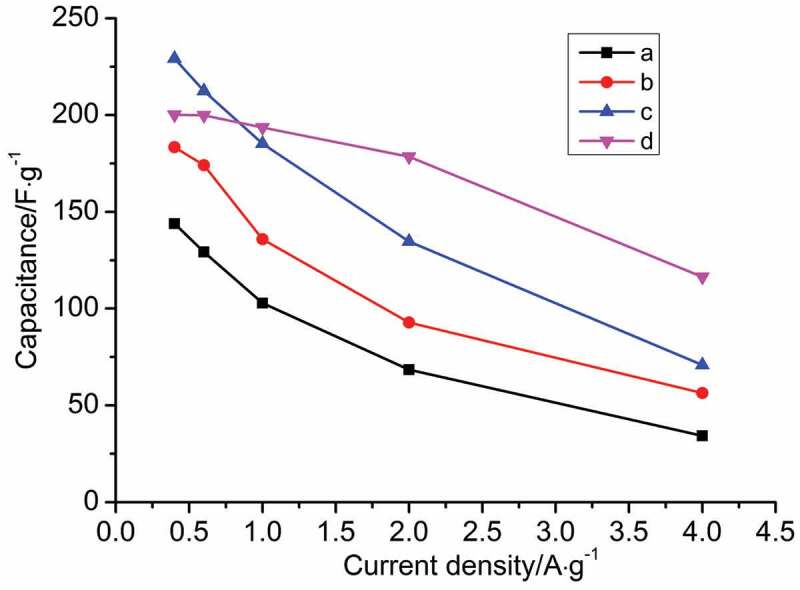
Specific capacitances of PANI as a function of current density prepared under the different conditions: (a) no salt; (b) NaCl; (c) MgSO_4_ and (d) AlCl_3._

The chronopotentiometry experiment was used to further investigate the effect of inorganic salts on the specific capacitance of PANI. Charge and discharge were carried out at different current densities (0.4, 0.6, 1.0, 2.0 and 4.0 A/g). The specific capacitance calculated according to the mass specific capacitance formula are shown in Figure 10. Compared with PANI synthesized without no salt in Figure 10a, the specific capacitance of PANI synthesized with inorganic salts decreases with the increase of current density. When the current density was 0.4 A/g, the specific capacitance of PANI synthesized with NaCl, MgSO_4_ and AlCl_3_ was the maximum, which was 180, 234 and 200 F/g, respectively. When the current density increased up to 4.0 A/g, the specific capacitance decreases to 75, 80 and 125 F/g. When the current density increased 10 times, the specific capacitance of PANI synthesized by AlCl_3_ decreased by about 37.5%, and the value of PANI prepared under other conditions significantly decreased. It is shown that inorganic salts have a significant effect on the electrochemical properties of PANI, and the increase of ionic strength of inorganic salt is helpful to improve the electrochemical properties of PANI.

## 4 Conclusion

PANI nanofibers were synthesized in the presence of inorganic salt, the influence of types and concentration of salts, doping acid and reaction temperature on PANI was studied through TEM, UV-vis and FTIR. The presence of D-CSA in the solution was conducive to form PANI nanofibers with small and smooth surface synthesized at the same condition; the moderate concentration of inorganic salt was helpful to adjust the diameter and the surface of PANI nanofibers at the different temperature; the electrochemical property of PANI increased with the increase of the ion strength; the influence of NaCl concentration on the nucleation of aniline polymerization was investigated through in situ UV-vis and ^1^H NMR, the reaction rate during the polymerization of aniline was reduced after the addition of NaCl at the low temperature.

## Supplementary Material

Supplemental Material
