# Supplementary material for: The effect of inorganic salt on the morphology and nucleation of polyaniline nanofibers synthesized via self-assembly
Source: Des Monomers Polym. 2023 Jan 18;26(1):45–53. doi: 10.1080/15685551.2023.2166727 (PMC9858426; doi:10.1080/15685551.2023.2166727)
Supplement: Supplemental Material [file TDMP_A_2166727_SM9234.doc]

Supporting information to “**The effect of inorganic salt on the morphology and nucleation of polyaniline nanofibers synthesized via self-assembly**”

Ruijuan Wang^*^, Yiqi Jing

Guangxi Key Lab of Agricultural Resources Chemistry and Biotechnology, Yulin Normal University, Yulin 537000, P. R. China


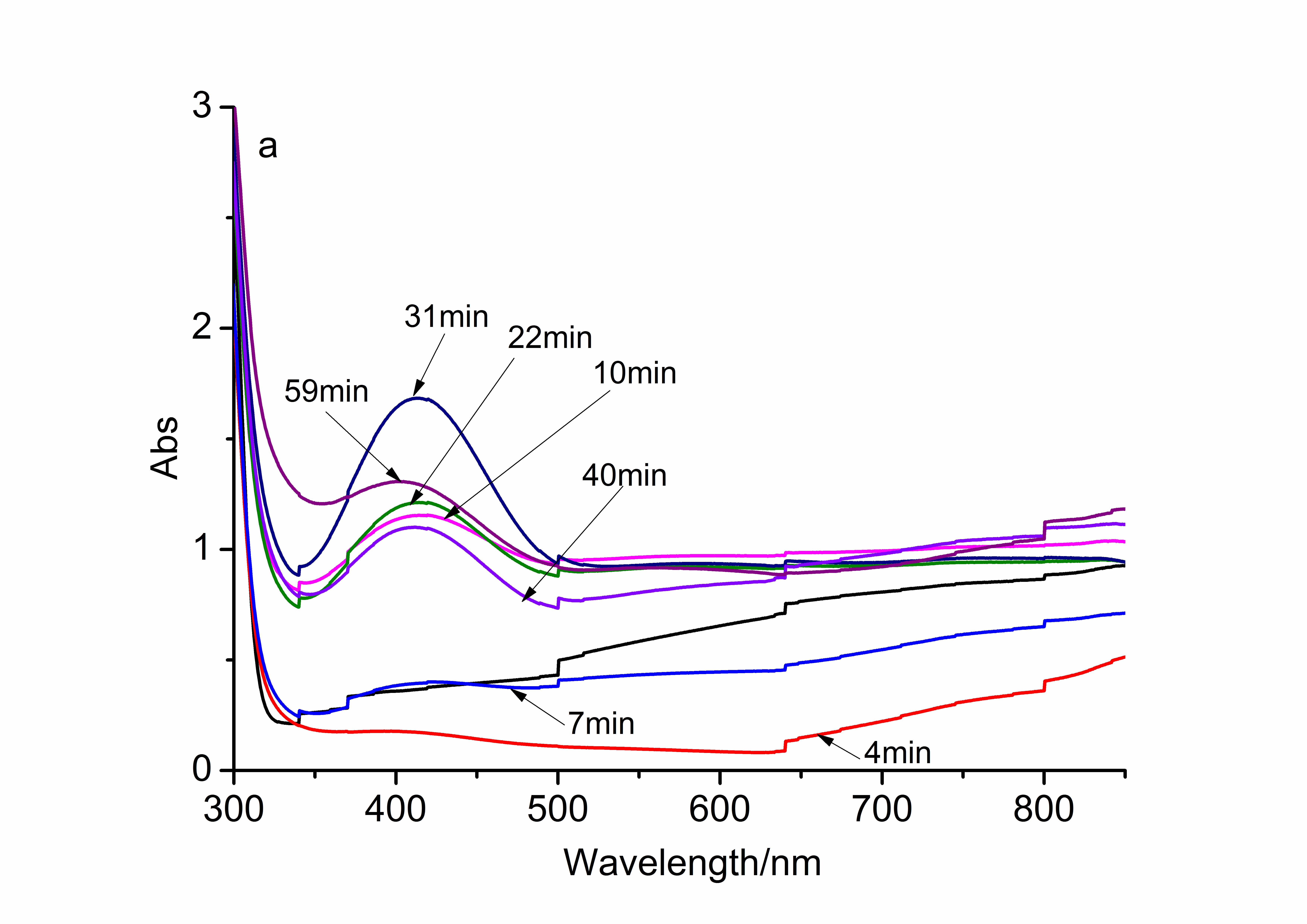

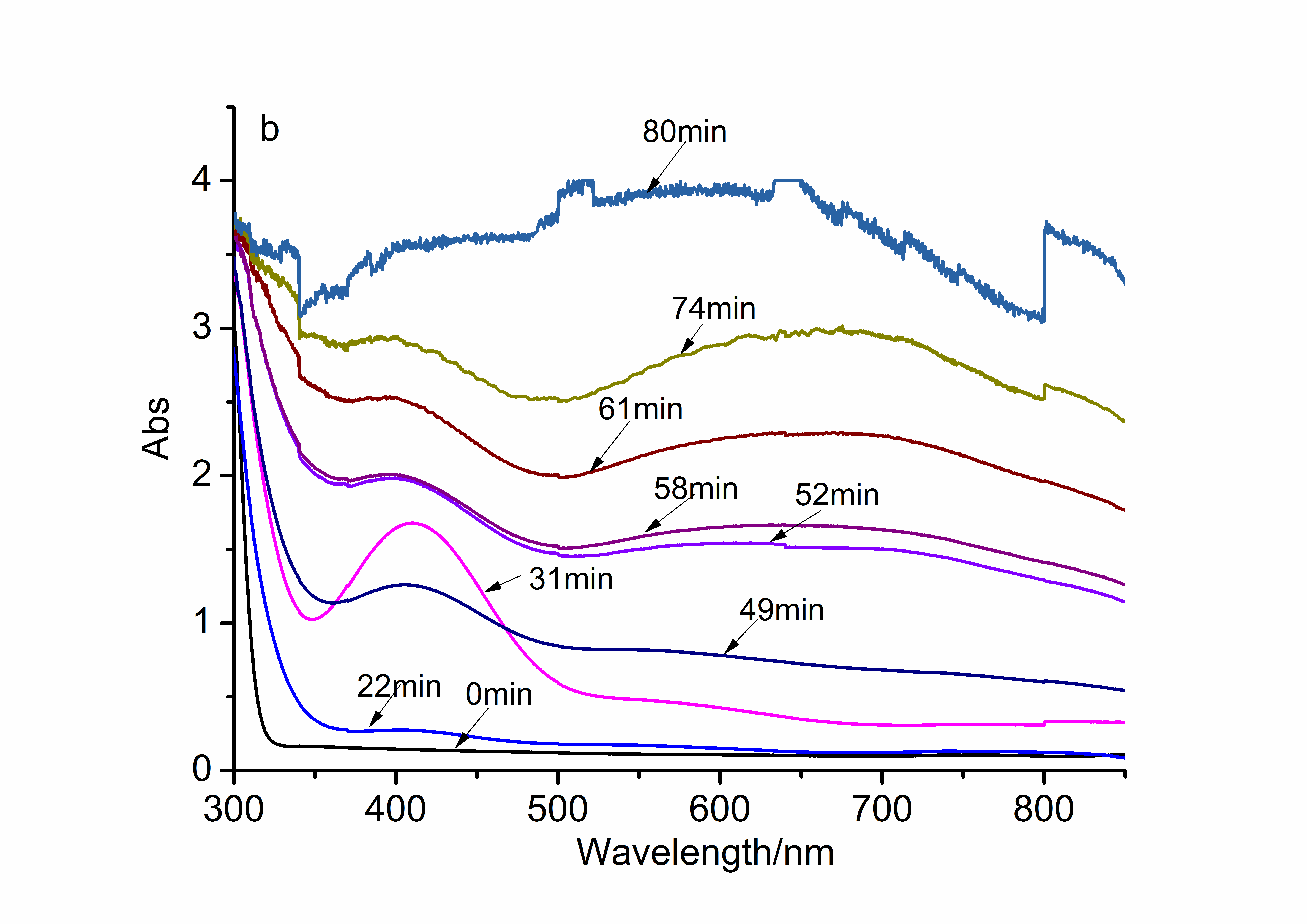


Figure S1 In situ Uv-vis spectra of PANI prepared at the different concentration of NaCl at 0°C, a) 0 M and b) 0.2 M


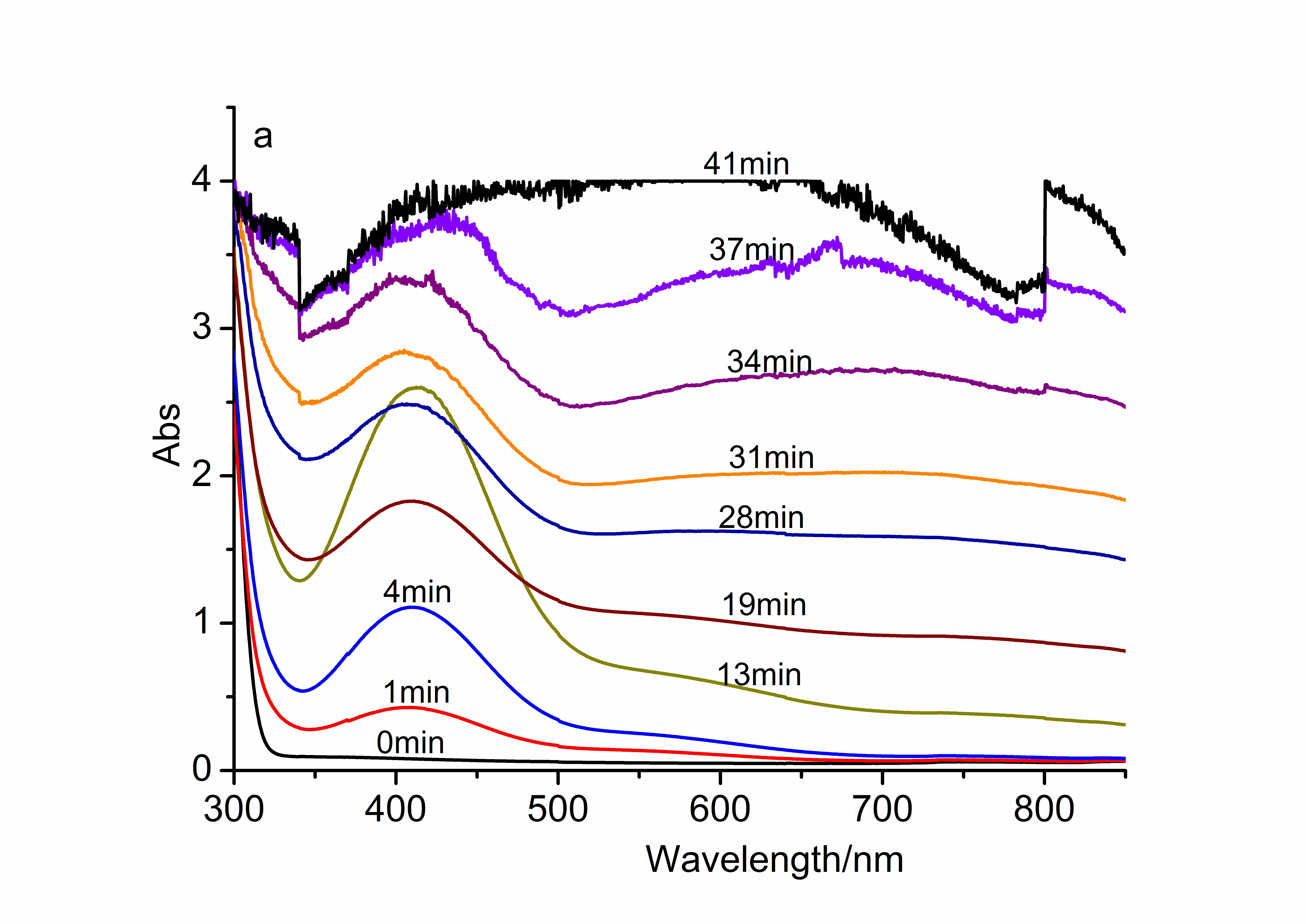

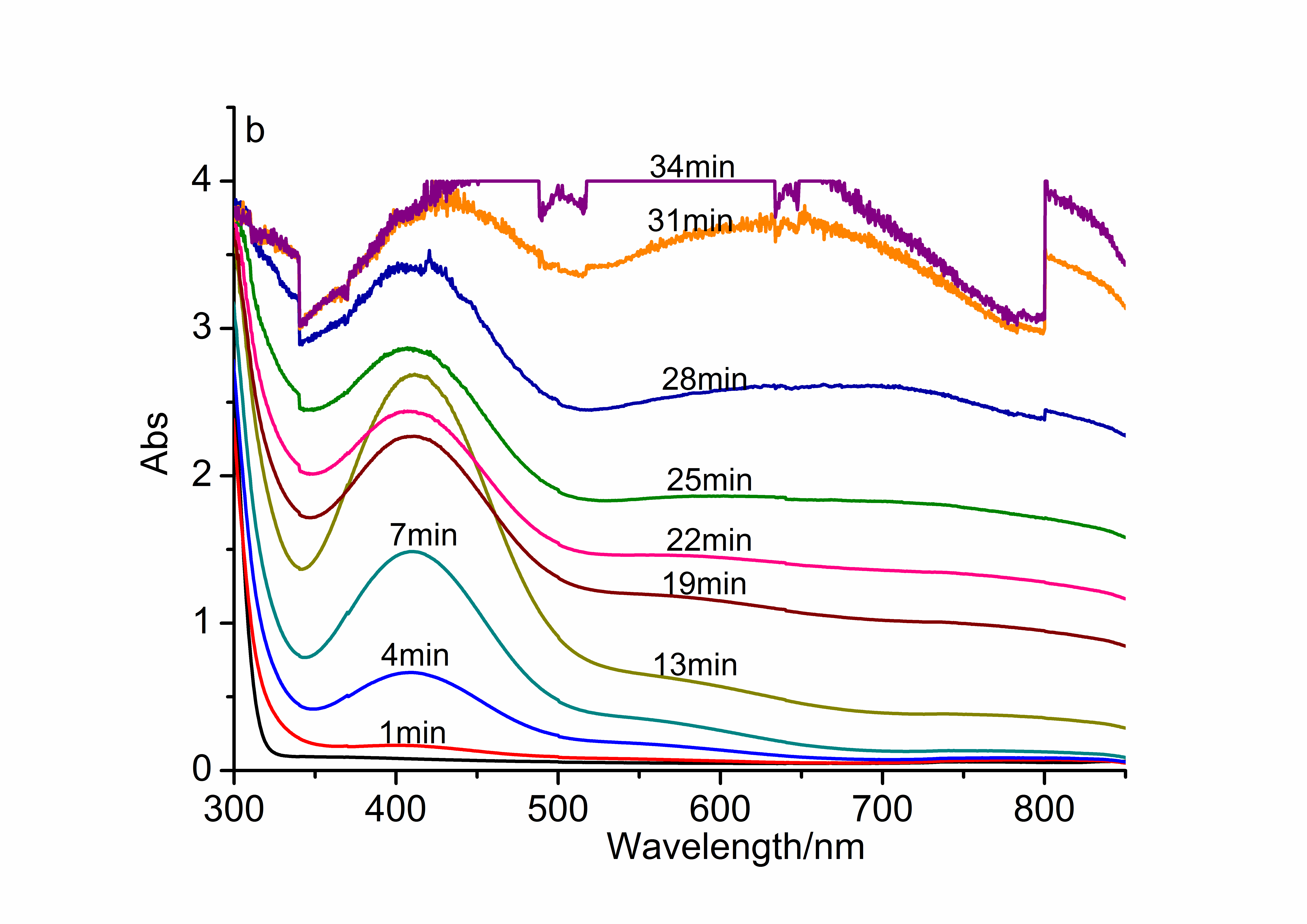


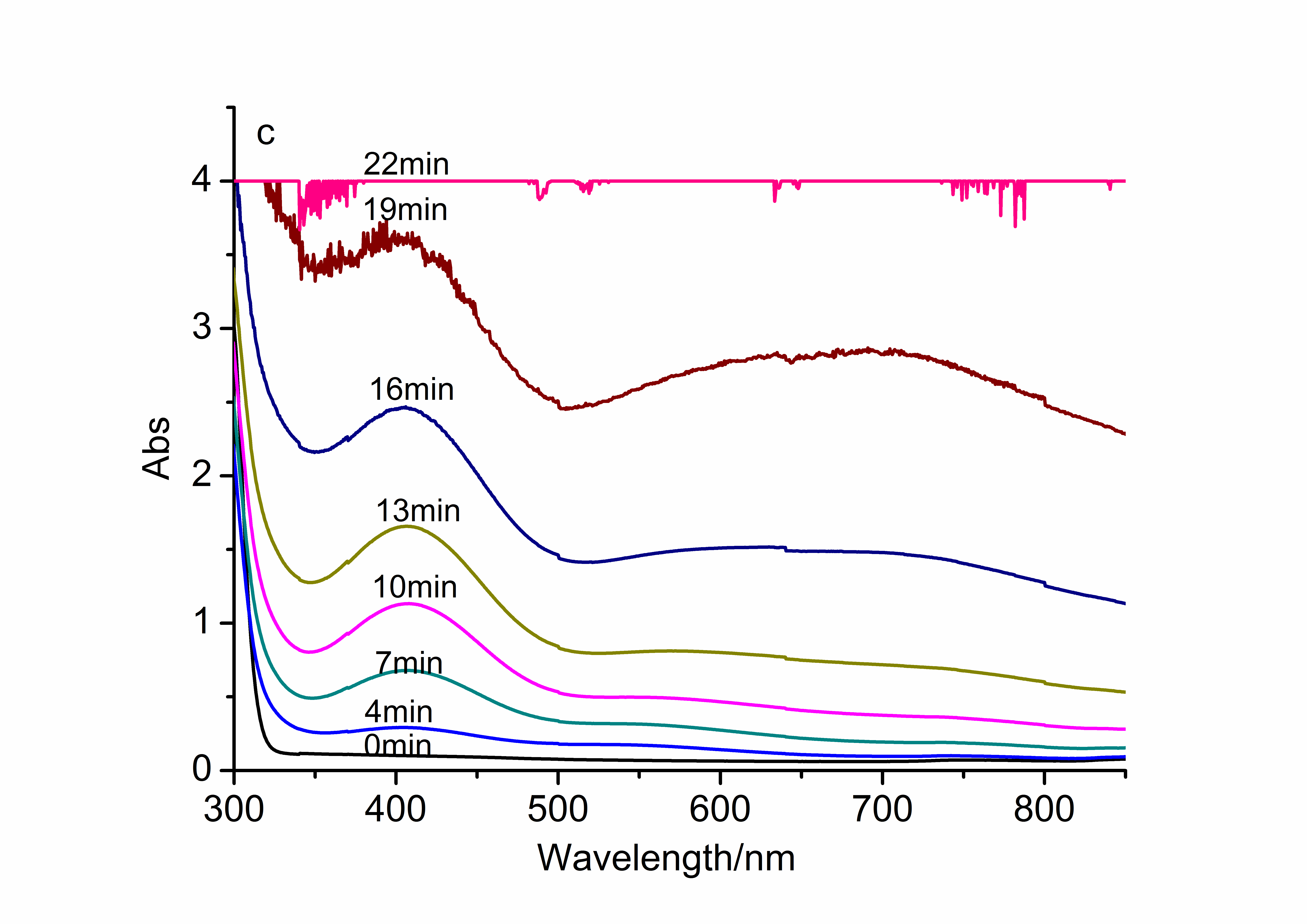


Figure S2 In situ Uv-vis spectra of PANI prepared at the different concentration of NaCl at 25°C, a) 0 M, b) 0.2 M and c) 0.4 M
